# Effectiveness of combined limonene and 4-hydroxyandrostenedione in the treatment of NMU-induced rat mammary tumours.

**DOI:** 10.1038/bjc.1994.170

**Published:** 1994-05

**Authors:** S. K. Chander, A. G. Lansdown, Y. A. Luqmani, J. J. Gomm, R. C. Coope, N. Gould, R. C. Coombes

**Affiliations:** Department of Oncology, Celltech Ltd, Slough, Berkshire, UK.

## Abstract

**Images:**


Br. J. Cancer (1994), 69, 879-882 © Macmillan Press Ltd., 1994

### Effectiveness of combined limonene and 4-hydroxyandrostenedione in the treatment of NMU-induced rat mammary tumours

S.K. Chander[1], A.G.B. Lansdown[2], Y.A. Luqmani[3], J.J. Gomm[3], R.C. Coope[3], N. Gould[4] & R.C. Coombes[3]

[1]*Department of Oncology, Celltech Ltd, 216 Bath Road, Slough, Berkshire SL1 4EN, UK; Departments of* [2]*Comparative Biology and* [3]*Medical Oncology, Charing Cross Hospital Medical School, Fulham Palace Road, London W6 8RE, UK;* [4]*Department of Human Oncology, University of Wisconsin-Madison, 600 Highland Avenue, K4/332 CSC, Madison, Wisconsin 53792, USA.*

**Summary** Limonene, a monocyclic monoterpene, occurs naturally in orange peel oil. It has been shown to exhibit both chemopreventive and chemotherapeutic activity without toxicity in rodent models. In this study we examined the effect of limonene both at maximally optimal and suboptimal doses and in combination with suboptimal doses of 4-hydroxyandrostrenedione on nitrosmethylurea-induced rat mammary tumours. A 10% limonene dose mixed in the diet caused tumour regression in all animals. A 5% limonene dose was only able to cause regression in 50% of the rats ($P < 0.05$). A suboptimal dose of 4-hydroxyandrostrenedione (12.5 mg kg$^{-1}$) resulted in tumour regression in 75% of rats. A combination of 5% limonene with 4-hydroxyandrostrenedione (12.5 mg kg$^{-1}$) resulted in a greater tumour regression (83.3%) than either agent given individually ($P < 0.001$ and 0.006 for limonene/4-hydroxyandrostrenedione *vs* limonene alone and 4-hydroxyandrostrenedione alone respectively).

The monocyclic monoterpene limonene is a natural product constituting up to 95% of orange peel oil and a considerable proportion of many other essential oils. Limonene has significant chemopreventive and chemotherapeutic activity without toxicity in rodents (Elegbede *et al.*, 1984, 1986; Elson *et al.*, 1988; Maltzman *et al.*, 1989; Wattenberg *et al.*, 1989, 1991). Most studies have been directed at the use of limonene in the prevention of carcinogen-induced cancer (Wattenberg *et al.*, 1983; Elegbede *et al.*, 1984). It has been shown that limonene inhibits rat mammary carcinomas induced by both the indirectly acting carcinogen DMBA (Elegbede *et al.*, 1984) and the directly acting carcinogen NMU (Maltzman *et al.*, 1989). The inhibitory effect of limonene was observed at both the initiation and promotion/progression stages for DMBA-induced cancers (Elson *et al.*, 1988), but only at the promotion/progression stage for NMU-induced mammary cancer (Maltzman *et al.*, 1989).

The suppressive activity of limonene during the promotion phase of rat mammary carcinogenesis has been demonstrated in both chemical carcinogen (Maltzman *et al.*, 1989) and *ras*-induced (Moore *et al.*, 1991) model systems. Rats fed a diet containing 5% limonene during the promotion phase only of the NMU model exhibited longer latency and approximately 5-fold fewer tumours than controls (Maltzman *et al.*, 1989).

It has been shown that limonene can selectively inhibit protein isoprenylation (Crowell *et al.*, 1991), a post-translational modification in which an isoprene group is covalently attached to the carboxy terminus. Most of the isoprenylated proteins affected by limonene have a molecular weight of 20,000-26,000 (Crowell *et al.*, 1991) and are small G-proteins such as the members of the p21-*ras* family (Maltese *et al.*, 1990).

The effectiveness of limonene alone on NMU-induced rats has already been shown by previous workers (Haag *et al.*, 1992). In this study the possibility of using limonene in combination with 4-hydroxyandrostenedione (4-HAD), a potent aromatase inhibitor, was investigated to explore whether the inhibitory activity of suboptimal doses of 4-HAD and limonene when given together could result in greater inhibition of NMU-induced tumour growth than either of these agents alone. Such an approach could open new avenues for using limonene in combination therapy.

Correspondence: S.K. Chander.
Received 10 November 1993; and in revised form 17 January 1994.

### Materials and methods

### In vivo *studies*

Inbred virgin female (Ludwig/Wistar/Olac) rats bearing tumours induced with NMU were supplied by Olac (Oxon, UK). These were used in the manner described previously (Wilkinson *et al.*, 1986). In all studies, adult rats bearing tumours between 10 and 20 mm in diameter were randomised. All rats were treated daily with appropriate drug for 4 weeks. Tumour measurements were made weekly for 4 weeks by measuring two diameters at right angles with vernier callipers. Tumour volume was estimated using the following formula:

$$\pi/6\ [(d_1 \times d_2)^{3/2}]$$

where $d_1$ and $d_2$ are the two diameters at right angles to each other.

At the end of the 4-week period, all palpable lesions were removed from the mammary area and stored in liquid nitrogen and subsequently used for histological examination.

### *Drug schedules*

*Limonene alone* Rat high-fat diet (SDS, UK) was powdered using a food processor and then the required amount of limonene (w/v), supplied by Aldridge (USA), was added to the powdered diet to give a final 10% limonene in the diet. The diet (prepared weekly) was placed in plastic bags and kept at −20°C until required. Rats were fed fresh diet daily (for 4 weeks) by placing in glass jars in the cages.

*4-Hydroxyandrostrendione alone* A suboptimal dose of 12.5 mg kg$^{-1}$ 4-HAD (Ciba Geigy) was resuspended in saline and formed a white suspension, which was administered daily at a dose of 12.5 mg kg$^{-1}$ (2.5 mg 0.1 ml$^{-1}$ per rat) subcutaneously for 4 weeks. A dose of 50 mg kg$^{-1}$ 4-HAD has previously been shown to be maximally effective (Wilkinson *et al.*, 1986).

*Combination therapy* Rats were given a 5% (suboptimal) dose of limonene in the diet as detailed above. 4-Hydroxyandrostrenedione in combination was given subcutaneously at a suboptimal dose of 12.5 mg kg$^{-1}$, daily for 4 weeks.

### *Histological studies*

At the end of the 4 week period of drug treatment animals were sacrificed and tumours were resected and immediately frozen in liquid nitrogen. Frozen sections (8 μm) were stained using standard haematoxylin and eosin, and examined for morphological changes.

### Results

### *Limonene treatment*

The effect of 10% limonene in the diet on the growth of NMU-induced tumours is shown in Figure 1. After 4 weeks' treatment, limonene produced tumour regression in all rats. Eighty-six per cent of the animals (13/15) exhibited greater than 50% tumour regression (Table I). Any new tumours that occurred after commencement of treatment also subsequently regressed in the limonene group. In the control group two (13.3%) rats showed tumour regression. Thus, limonene treatment produced a significantly beneficial response compared with control (Mann-Whitney $U = 1$, $P < 0.05$). We observed no weight loss at this dose of limonene.

### *Limonene in combination with 4-hydroxyandrostenedione*

The combination of suboptimal doses of 4-HAD with a suboptimal dose of limonene (5%) was compared and the results are shown in Figure 2. A 5% limonene dose only produced overall tumour regression of 50% (6/12) of rats,

**Figure 1** The effect of limonene on the growth of NMU-induced rat mammary tumours. Limonene was mixed into powdered diet to give a final 10% in diet and rats were fed, with daily changes, for 4 weeks. Tumour measurements were made weekly and the tumour volume calculated as described in Materials and methods. Results are expressed as the percentage change from the starting tumour volume. Error bars represent s.e.m.

**Table I** Effect of 10% limonene on the growth of NMU-induced rat mammary tumours

| | *Groups* Limonene (10% diet) | *Groups* Control |
|---|---|---|
| No. of rats | 15 | 15 |
| No. of new tumours | 1 | 0 |
| No. of rats showing < 50% regression | 2 | 2 |
| No. of rats showing > 50% regression | 13 | 0 |
| No. of rats showing progression | 0 | 13 |
| Percentage of rats showing > 50% regression | 86.6 | 0 |
| Percentage of rats showing any regression | 100 | 13.3[a] |

Rats were given limonene or vehicle daily for 4 weeks. Tumour growth was recorded every week for the 4-week period, by measuring two diameters at right angles with vernier callipers and tumour volume was estimated as described in Materials and methods. Each of the animals was categorised into one of three groups: (a) 50% or greater tumour regression; (b) 0–50% tumour regression; (c) tumour progression. [a]Mann-Whitney $U = 1$, $P < 0.05$.

but only 25% of animals showed > 50% tumour regression. 4-HAD administered alone caused regression in 75% (9/12) of rats. Moreover, 58% (7/12) of rats showed > 50% tumour regression. However, in combination, limonene plus 4-HAD caused regression in 83.3% (10/12) of rats, and this level of response was significantly greater than that achieved with the individual agents alone (*P*-values as given in Table II). The weights of rats were also recorded throughout the experiment and there was no significant loss compared with controls [mean weights in g (± s.d.) after 4 weeks: control = 198 ± 18, limonene alone = 194 ± 19; 4-HAD alone = 207 ± 12; limonene + 4-HAD = 206 ± 9].

### *Histological studies*

*Control group* Eight representative excised mammary tumours were examined. One consisted of loosely packed adenoid tissue with fibrous tissue separating acinar tissue. Mitoses were not seen frequently and cellular characteristics showed this tumour to be a benign fibroadenoma. The other seven tumours were classified as adenocarcinoma. They exhibited characteristic cellular pleomorphism, abundant mitoses and prominent papillary growth (Figure 3). Epithelia were commonly multilaminate. In some tumours, large acinar cavities or cysts were present and containing variable amounts of cell debris. Four of the tumours were well-differentiated carcinomas; their cellularity was more intense and papillary tufts prominent.

*Limonene-treated group* Two tumours out of 15 examined were quite benign. They contained some large debris-filled cavities, showed no evidence of proliferative activity and consisted of acinar tissue interspersed with connective tissue characteristic of adenocarcinoma, but for the most part the tissue was hypocellular, fragmentary and regressive (Figure 4). Some residual evidence of malignancy in the form of papillary tufts with pleomorphic cells and hypocellularity was seen, but mitotic activity was very low. These tumours exhibited fragmentary epithelia and were generally regressive.

Those rats treated with the combination of limonene and 4-HAD also showed similar characteristics to the limonene-treated groups.

### Discussion

Data presented in this study support previous findings that a 10% limonene dose in the diet is sufficient to cause tumour regression (Haag *et al.*, 1992); limonene showed regression in

**Figure 2** The effect of (suboptimal dose) 5% limonene and 12.5 mg $kg^{-1}$ 4-HAD administered separately and in combination on the growth of NMU-induced rat mammary tumours. Rats were treated daily for 4 weeks and tumour measurements made weekly. (□) control, (○) 5% limonene alone in diet, (●); 4-HAD alone given s.c., (■) 5% limonene (in diet) plus 4-HAD given s.c. Error bars represent s.e.m.

**Table II** Effect of 5% limonene/4-HAD on NMU-induced rat mammary tumours

| | Treatment groups | | | |
|---|---|---|---|---|
| | *Control* | *5% limonene* | *4-HAD (12.5 mg kg⁻¹)* | *5% limonene + 4-HAD* |
| No. of rats | 12 | 12 | 12 | 12 |
| No. of initial tumour (≥10 mm) | 20 | 19 | 14 | 16 |
| No. of new tumours | 2 | 0 | 1 | 0 |
| No. of rats showing <50% regression | 1 | 2 | 2 | 0 |
| No. of rats showing >50% regression | 0 | 4 | 7 | 10 |
| No. of rats showing progression | 11 | 6 | 3 | 2 |
| Percentage of rats showing >50% regression | 0.0 | 33.3 | 58.3 | 83.3 |
| Percentage of rats showing any regression | 8.3 | 50.0 | 75 | 83.3[a] |

Rats were given either drug or vehicle (as described in Materials and methods) daily for 4 weeks. Tumour growth was recorded every week over the 4 week period, by measuring two diameters at right angles with vernier callipers, and tumour volume was estimated using the formula given in Materials and methods. Animals were categorised into three groups: (a) 50% or greater tumour regression; (b) 0–50% tumour regression; (c) tumour progression. [a]Mann–Whitney *U*-test: limonene + 4-HAD *vs* control, $P < 0.001$; limonene + 4-HAD *vs* limonene, $P < 0.001$; limonene + 4-HAD *vs* 4-HAD, $P = 0.006$.

**Figure 3** Frozen section (stained with haematoxylin and eosin) of an NMU-induced rat mammary adenocarcinoma. Note dense cellular mass, papilliform growths and multilaminate epithelia. Magnification × 65.

**Figure 4** Haematoxylin and eosin-stained frozen section of NMU-induced rat mammary tumour illustrating fragmentary epithelia in a mammary tumour mass after treatment with limonene. Magnification × 130.

100% of rats, and 86.6% of rats showed >50% regression from the initial tumour volume. The histological studies showed that treatment with limonene caused regression of tumour mass, as most of the tissue showed regressive fragmentary epithelia.

The most significant finding was that suboptimal doses of an aromatase inhibitor, 4-HAD, could be used in combination with a suboptimal dose of limonene to produce regression in rats similar to that obtained with maximally effective doses of either limonene or 4-HAD separately. This suggests that the full potential of limonene in the treatment of breast cancer can be realised in a combination therapy minimising risk of toxicity, which might occur if higher doses were used in chronic treatment.

The mode of action of 4-HAD has been well studied (Coombes *et al.*, 1984) but the precise mechanism of action of limonene still remains to be elucidated. Crowell *et al.* (1991) recently reported that, in cultured fibroblasts and mammary epithelia, limonene and its major circulating metabolites selectively inhibited the isoprenylation of cellular proteins, in particular those in the molecular weight range of 20,000–26,000. Most of these are small G-proteins that are likely to be involved in signal transduction (Maltese *et al.*, 1990). Isoprenylation of these proteins involves the covalent addition of either farnesyl or geranyl–geranyl moieties to the carboxyl end of the protein. Inhibition of this hydrophobic post-translational modification prevents the protein from assuming its correct subcellular location, thus interfering with its function. Haag *et al.* (1992) speculated that this very selective but partial inhibition of this isoprenylation of small G-proteins may be involved with the regression of certain tumours, and therefore this process could be a potentially useful target for cancer therapeutic strategies.

Crowell *et al.* (1991) have recently shown that there are more potent monoterpene inhibitors of protein isoprenylation than limonene, such as perillic and dihydroperillic acid, which are found as active metabolites in rats (Crowell *et al.*, 1991). These could potentially be used in smaller doses than limonene. It will also be useful to extend this *in vivo* evaluation of limonene and similarly active agents to other tumour types in addition to mammary carcinomas.

**Abbreviations:** 4-HAD, 4-hydroxyandrostenedione; NMU, nitrosmethylurea; DMBA, dimethylbenz[*a*]anthracene.

### References

COOMBES, R.C., GROSS, P., DOWSETT, M., GAZET, J.-C. & BRODIE, A. (1984). 4-Hydroxyandrostenedione in treatment of postmenopausal patients with advanced breast cancer. *Lancet*, **ii**, 1237–1239.

CROWELL, P.L., CHANG, R.R., REN, Z., ELSON, C.E. & GOULD, M.N. (1991). Selective inhibition of isoprenylation of 21–26 kDa proteins by the anticarcinogen d-limonene and its metabolites. *J. Biol. Chem.*, **266**, 17679–17685.

ELEGBEDE, J.A., ELSON, C.E., QURESHI, A., TANNER, M.A. & GOULD, M.N. (1984). Inhibition of DMBA-induced mammary cancer by the monoterpene d-limonene. *Carcinogenesis*, **5**, 661–664.

ELEGBEDE, J.A., ELSON, C.E., TANNER, M.A., QURESHI, A. & GOULD, M.N. (1986). Regression of rat primary mammary tumours following dietary d-limonene. *J. Natl Cancer Inst.*, **76**, 323–325.

ELSON, C.E., MALTZMAN, T.H., BOSTON, J.L., TANNER, M.A. & GOULD, M.N. (1988). Anti-carcinogenic activity of d-limonene during the initiation and promotion/progression stages of DMBA-induced rat mammary carcinogenesis. *Carcinogenesis*, **9**, 331–332.

HAAG, J.D., LINDSTROM, M.J. & GOULD, M.N. (1992). Limonene-induced regression of mammary carcinomas. *Cancer Res.*, **52**, 4021–4026.

MALTESE, W.A., SHERIDAN, K.M., REPKO, E.M. & ERDMAN, R.A. (1990). Post-translational modification of low molecular mass GTP-binding proteins by isoprenoid. *J. Biol. Chem.*, **265**, 2148–2155.

MALTZMAN, T.H., HURT, L.M., ELSON, C.E., TANNER, M.A. & GOULD, M.N. (1989). The prevention of nitrosomethylurea-induced mammary tumours by d-limonene and orange oil. *Carcinogenesis*, **10**, 781–783.

MOORE, C.J., KENNAN, W.S., WANG, B.C. & GOULD, M.N. (1991). Inhibition of *ras*-induced rat mammary carcinogenesis by limonene (abstract 782). *Proc. Am. Assoc. Cancer Res.*, **32**, 131.

WATTENBERG, L.W. (1983). Inhibition of neoplasia by minor dietary constituents. *Cancer Res.*, **43**, 2448s–2453s.

WATTENBERG, L.W. (1989). Inhibition of N-nitrosodiethylamine carcinogenesis in mice by naturally occurring organosulphur compounds and monoterpenes. *Cancer Res.*, **49**, 2689–2692.

WATTENBERG, L.W. & COCCIA, J.B. (1991). Inhibition of 4-(methylnitrosamino)-1-(3-pyridyl)-1-butanone carcinogenesis in mice by d-limonene and citrus oils. *Carcinogenesis*, **12**, 115–117.

WILKINSON, J.R., WILLIAM, J.C., SINGH, D., GOSS, P.E., EASTON, D. & COOMBES, R.C. (1986). Response of nitrosomethylurea-induced rat mammary tumours to endocrine therapy and comparison with clinical response. *Cancer Res.*, **46**, 4862–4865.


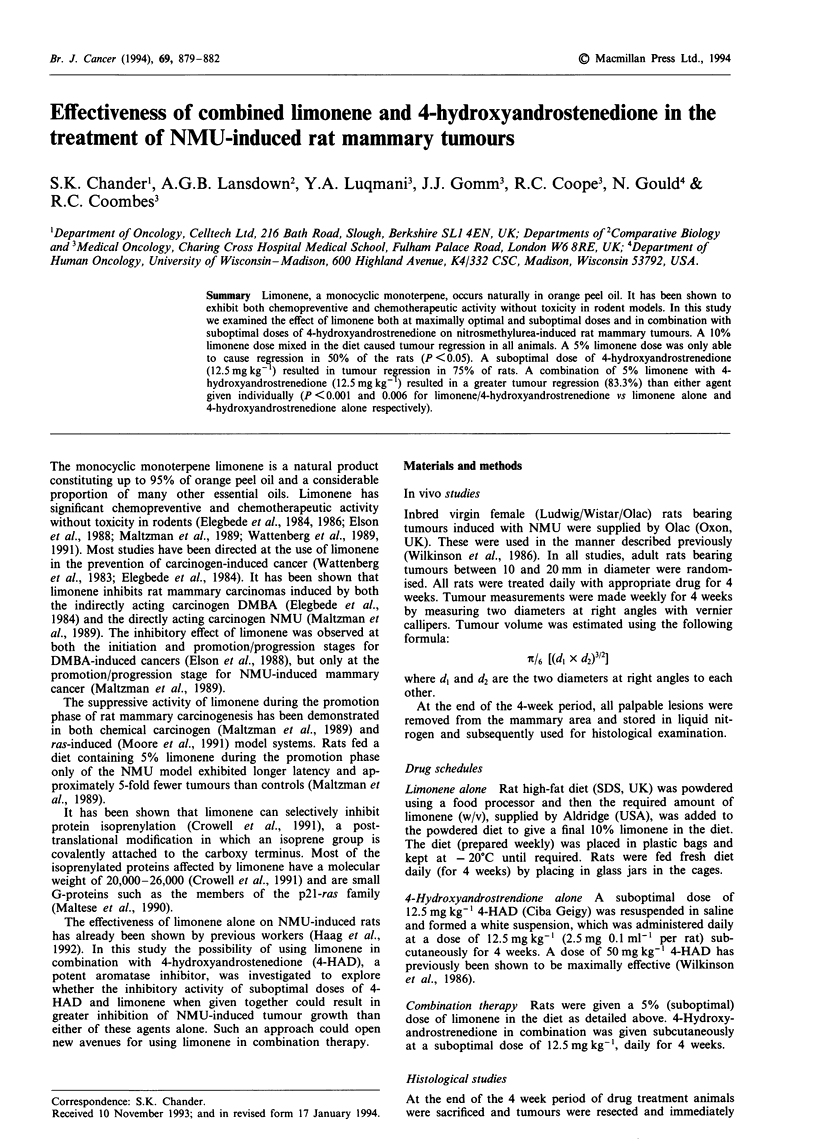

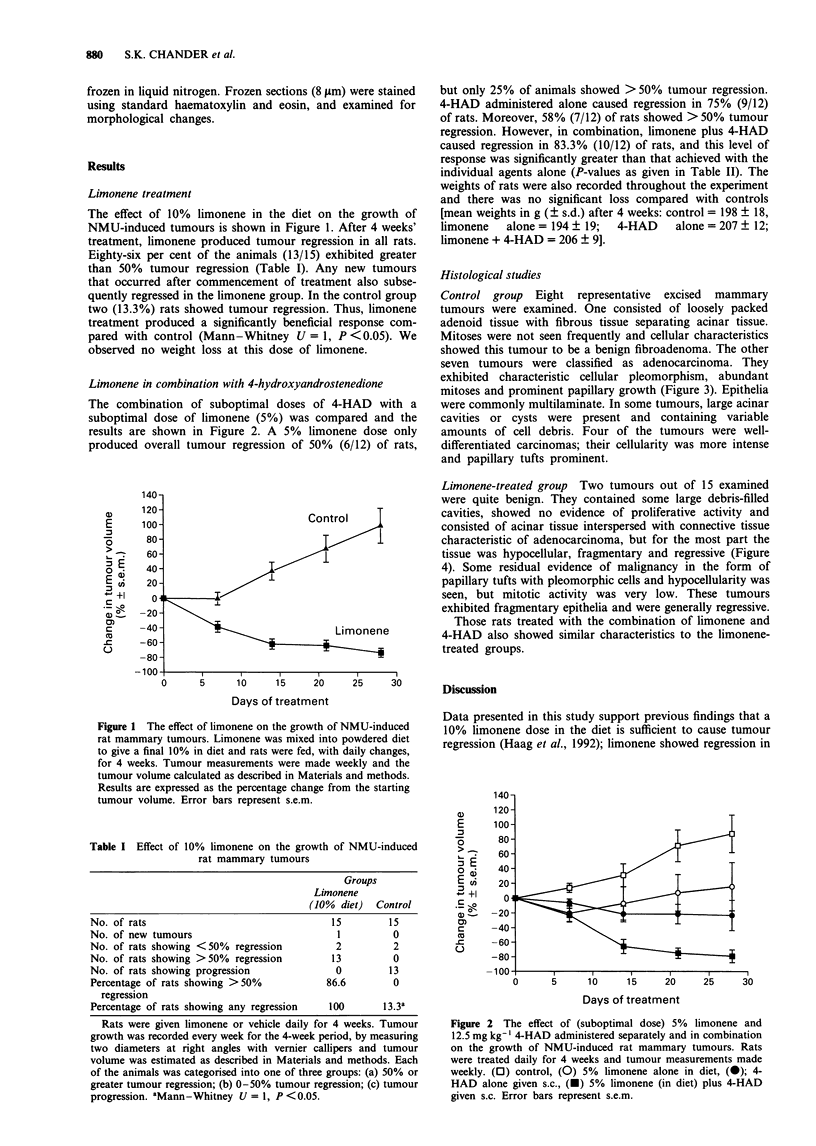

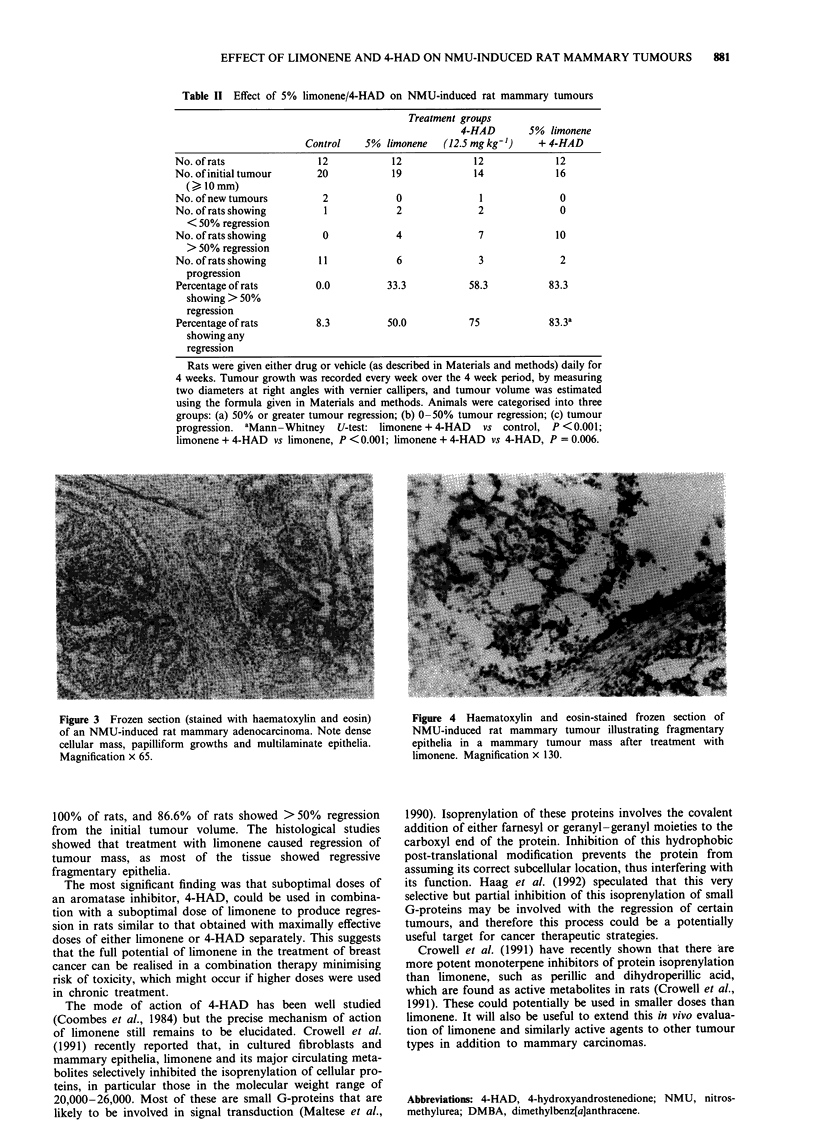

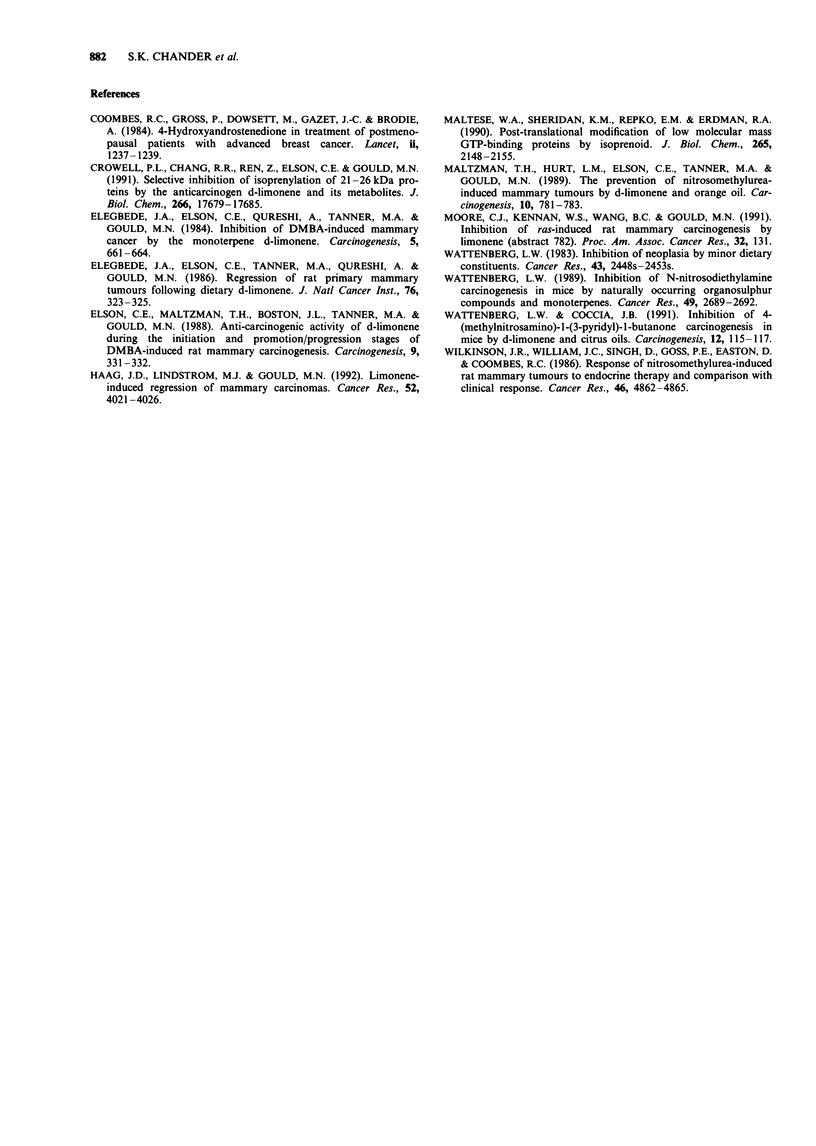


## References

[OCR_00396] Coombes R. C., Goss P., Dowsett M., Gazet J. C., Brodie A. (1984). 4-Hydroxyandrostenedione in treatment of postmenopausal patients with advanced breast cancer.. Lancet.

[OCR_00402] Crowell P. L., Chang R. R., Ren Z. B., Elson C. E., Gould M. N. (1991). Selective inhibition of isoprenylation of 21-26-kDa proteins by the anticarcinogen d-limonene and its metabolites.. J Biol Chem.

[OCR_00408] Elegbede J. A., Elson C. E., Qureshi A., Tanner M. A., Gould M. N. (1984). Inhibition of DMBA-induced mammary cancer by the monoterpene d-limonene.. Carcinogenesis.

[OCR_00414] Elegbede J. A., Elson C. E., Tanner M. A., Qureshi A., Gould M. N. (1986). Regression of rat primary mammary tumors following dietary d-limonene.. J Natl Cancer Inst.

[OCR_00420] Elson C. E., Maltzman T. H., Boston J. L., Tanner M. A., Gould M. N. (1988). Anti-carcinogenic activity of d-limonene during the initiation and promotion/progression stages of DMBA-induced rat mammary carcinogenesis.. Carcinogenesis.

[OCR_00427] Haag J. D., Lindstrom M. J., Gould M. N. (1992). Limonene-induced regression of mammary carcinomas.. Cancer Res.

[OCR_00432] Maltese W. A., Sheridan K. M., Repko E. M., Erdman R. A. (1990). Post-translational modification of low molecular mass GTP-binding proteins by isoprenoid.. J Biol Chem.

[OCR_00438] Maltzman T. H., Hurt L. M., Elson C. E., Tanner M. A., Gould M. N. (1989). The prevention of nitrosomethylurea-induced mammary tumors by d-limonene and orange oil.. Carcinogenesis.

[OCR_00457] Wattenberg L. W., Coccia J. B. (1991). Inhibition of 4-(methylnitrosamino)-1-(3-pyridyl)-1-butanone carcinogenesis in mice by D-limonene and citrus fruit oils.. Carcinogenesis.

[OCR_00448] Wattenberg L. W. (1983). Inhibition of neoplasia by minor dietary constituents.. Cancer Res.

[OCR_00452] Wattenberg L. W., Sparnins V. L., Barany G. (1989). Inhibition of N-nitrosodiethylamine carcinogenesis in mice by naturally occurring organosulfur compounds and monoterpenes.. Cancer Res.

[OCR_00461] Wilkinson J. R., Williams J. C., Singh D., Goss P. E., Easton D., Coombes R. C. (1986). Response of nitrosomethylurea-induced rat mammary tumor to endocrine therapy and comparison with clinical response.. Cancer Res.

